# A systematic review on the role of the endoscope in the surgical management of cerebellopontine angle tumors: is it time to draw the conclusion?

**DOI:** 10.1007/s00405-025-09427-4

**Published:** 2025-04-30

**Authors:** Raffaele De Marco, Sébastien Froelich, Andrea Albera, Diego Garbossa, Francesco Zenga

**Affiliations:** 1 Department of Neuroscience “Rita Levi Montalcini”, University of Turin, Via Cherasco, 15, Turin, 10126 Italy; 2 Department of Neurosurgery, Lariboisière Hospital, Paris, France; 3 Pituitary and Skull Base Surgery Unit, “Città della Salute e della Scienza” University Hospital, Turin, Italy; 4 Division of Otorhinolaryngology, Department of Surgical Sciences, University of Turin, Turin, Italy

**Keywords:** Vestibular schwannoma, Epidermoid tumor, Meningioma, Endoscope, Facial nerve

## Abstract

**Objective:**

The use of the endoscope has brought major changes in skull base surgery in the last decades. In the cerebellopontine angle (CPA), it has shown few advantages over microscopic surgery alone, evolving towards a full-endoscopic surgery for neurovascular conflicts and tumors. This review aims to systematically analyze the literature about the use of the endoscope in the cerebellopontine angle tumors.

**Methods:**

Pubmed/Medline and Embase databases were investigated applying PRISMA guidelines without time restrictions to find all adult patients affected by an extra-axial cerebellopontine angle tumor (vestibular schwannoma, meningioma, epidermoid tumor, or other extra-axial lesions) treated using only the endoscope (full-endoscopic, FE or endoscopic-controlled, EC) or with endoscopic assistance (EA).

**Results:**

After article selection, a total of 2489 patients have been treated for a CPA lesion using the endoscope: 2054 vestibular schwannomas (VS), 368 epidermoid tumors (ET), 41 meningiomas and 26 among other pathologies. The retrosigmoid approach was the most frequently employed surgical corridor, irrespective of lesion type, for both full-endoscopic and endoscopic-assisted procedures. Although a great heterogeneity should be highlighted among the selected series of VS (1539), a weighted average of 92.5% of gross total resection (GTR) was obtained and 90% out of 1332 showed a good facial nerve outcome when comparable. Advantages in term of recognition of residuals have been described for the CPA meningiomas and multicompartmental epidermoid tumors with origin from CPA cistern, without increasing the risk of complications.

**Conclusions:**

Despite different accepted advantages, the number of tumors in which the endoscope has been included among the surgical armamentarium is still limited compared to the number of the full-microscopic resections. After almost 30 years since its value was recognized, the number of prospective and case-control studies is still scarce to affirm a real benefit leading to its routinary use.

**Supplementary Information:**

The online version contains supplementary material available at 10.1007/s00405-025-09427-4.

## Introduction

The use of the endoscope in pathologies of the posterior cranial fossa is not a novel phenomenon. However, its utilization has undergone a significant evolution over the past decades, progressing from a supplementary visualization device (endoscope-assistance, EA) to the sole visualization instrument during surgical procedures (endoscope-controlled, EC or full-endoscopic, FE).

This resulted in a visualization that extended beyond the corners, which are unavoidable in the cerebellopontine angle (CPA) due to the incidence of the surgical microscope’s light beam and the anatomical conformation of the region. It immediately demonstrated its advantages in the context of tumor pathology of the CPA [1, 2]. With a superior view of cranial nerves’ junctions at brainstem, their dural exit and their vascular relationships [3], the endoscope has been demonstrated to offer also other advantages such as small craniotomies that respect the mini-invasiveness concept, residual tumor visualization, and easy recognition of opened mastoid cells. Nevertheless, the majority of neurosurgeons are less familiar with endoscopic surgery than microscopic surgery, which remains the gold standard, and its routine application is limited to a few centers. The objective of this literature review is to investigate the dissemination and outcomes of this instrument (both EA, EC, and FE) in the management of extra-axial pathologies affecting the CPA.

## Materials and methods

The Medline/Pubmed and Embase databases were queried with different combinations of terms indicating posterior fossa and specifically cerebellopontine angle lesions treated by using the endoscope (cerebellopontine angle tumors, vestibular schwannoma, posterior fossa skull base meningioma, posterior fossa epidermoid cyst AND endoscopic surgery OR resection), regardless of whether it was used to control or directly contribute to tumor resection. The search was performed in November 2024 following PRISMA Guidelines and no time restriction was considered (see supplementary materials). Only peer-reviewed articles in English were included in the analysis.

The literature was systematically reviewed by two independent reviewers (RDM and DG). All disagreements were resolved by further discussion with another contributing author (FZ).

All adult patients (> 18 years old) with a tumoral extra-axial pathology of the CPA angle treated using an endoscope (as assistance or as a unique magnification instrument in controlled or fully endoscopic resection) were considered in the results. The following information was collected: year of publication, institution, type of study, type of approach, endoscope type (rigid versus flexible), use of endoscope holder, type(s) of tumor (only extra-axial tumors), dimensions, other classification assessing tumor size in vestibular schwannoma or involvement and/or extension to different compartments in case of epidermoid tumors, surgical time, extent of resection, length of stay, surgical complications and specifically those caused by endoscope introduction or movements, postoperative facial nerve (FN) outcome and/or auditory function. The availability of all or part of this information was considered among inclusion criteria. The absence of information regarding extent of resection or cranial nerves’ functions were considered, on the other hand, as exclusion criteria.

Anatomic/cadaveric studies were excluded, while case reports and small case series (< 5 patients) were contemplated to obtain the total number of CPA lesions where the endoscope was used, but they were excluded from the ultimate analysis (specifically from tabulation), in order to have a sufficient power analysis for comparison between studies. Descriptive statistics were reported with mean and standard deviation for continuous variables and with frequency and percentage for ordinal and nominal variables.

The Newcastle-Ottawa Scale (NOS) was applied to assess the quality of the selected studies. Specifically, the postoperative function of the facial nerve was considered as an outcome and, when specified, the postoperative auditory function was also taken into consideration. A study met the representativeness criterion if the population included at least 10 patients. The cohort should have been characterized by lesions of different sizes. Being mostly single-arm observational studies, the comparability section was not considered in the final count. The description of the operative technique and more precisely the use of the endoscope during surgery satisfied the criterion of exposure.

A description of initial symptoms was required to exclude any preoperative impairment of the facial nerve function. The House-Brackmann scale for postoperative FN function had to be used for grading. The outcome was considered acceptable if reported as individual grades or by dividing two classes: favorable outcomes for FN function were ≤ 2 and unfavorable when HB ≥ 3. A star was assigned when at least 95% of selected patients were followed for at least 12 months. Studies rated four stars without this requirement were also analyzed, considering the potential harm of the endoscope. This is due to the perceived influence of the endoscope on facial nerve function, particularly in the short term [4].

All the statistical analysis was conducted using an open-source software built on R language (The jamovi project (2021)”. jamovi. (Version 2.6) [Computer Software]. Retrieved from https://www.jamovi.org). Statistical significance was set at *p* ≤ 0.05. A meta-analysis of proportion was conducted on an online open-source software [5].

## Results

**Fig. 1:**
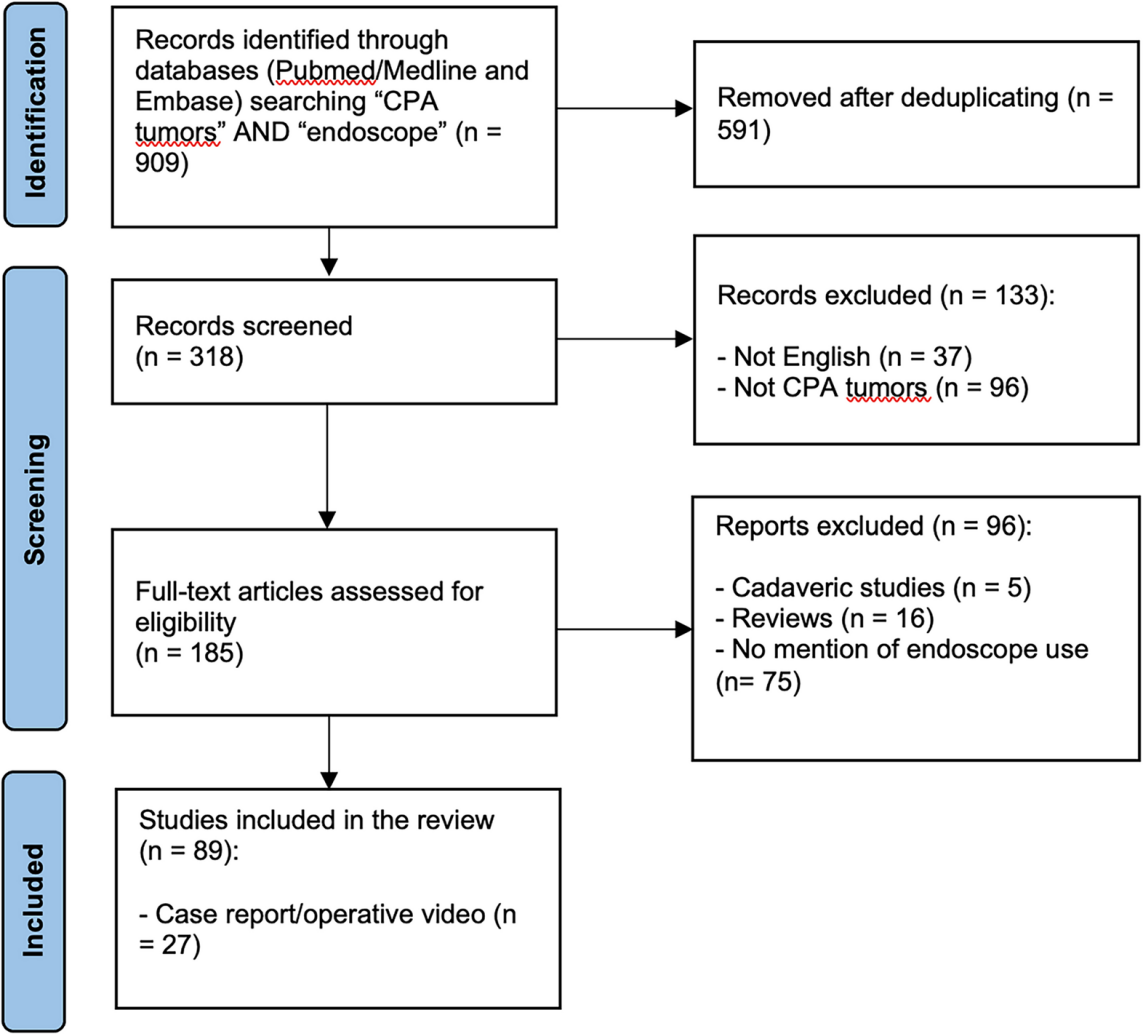
PRISMA flowchart

Nine hundred and nine articles were obtained from Pubmed/Medline and Embase databases after combining the results of each string (Supplementary material); 591 were excluded after deduplicating, 37 because they were not in English, and 96 articles were out of topic. For those where treatment of a posterior fossa lesion has been reported, 75 did not use the endoscope, 16 were reviews and 5 were cadaveric studies. In the end, only 87 articles were considered for full-text analysis. Only articles with more than 5 patients were screened for all information and selected for tabulation (Fig. 1).

A total number of 2,489 patients affected by a CPA lesion (vestibular schwannoma, meningioma, epidermoid tumor or other extra-axial pathologies with involvement of the cerebellopontine angle, such as cholesteatoma) has been treated by using the endoscope as additional magnification tool for inspection (EA) or as the only optical instrument (EC) under continuous microscope view or throughout the surgical procedure (FE) without the use of the microscope.

**Fig. 2:**
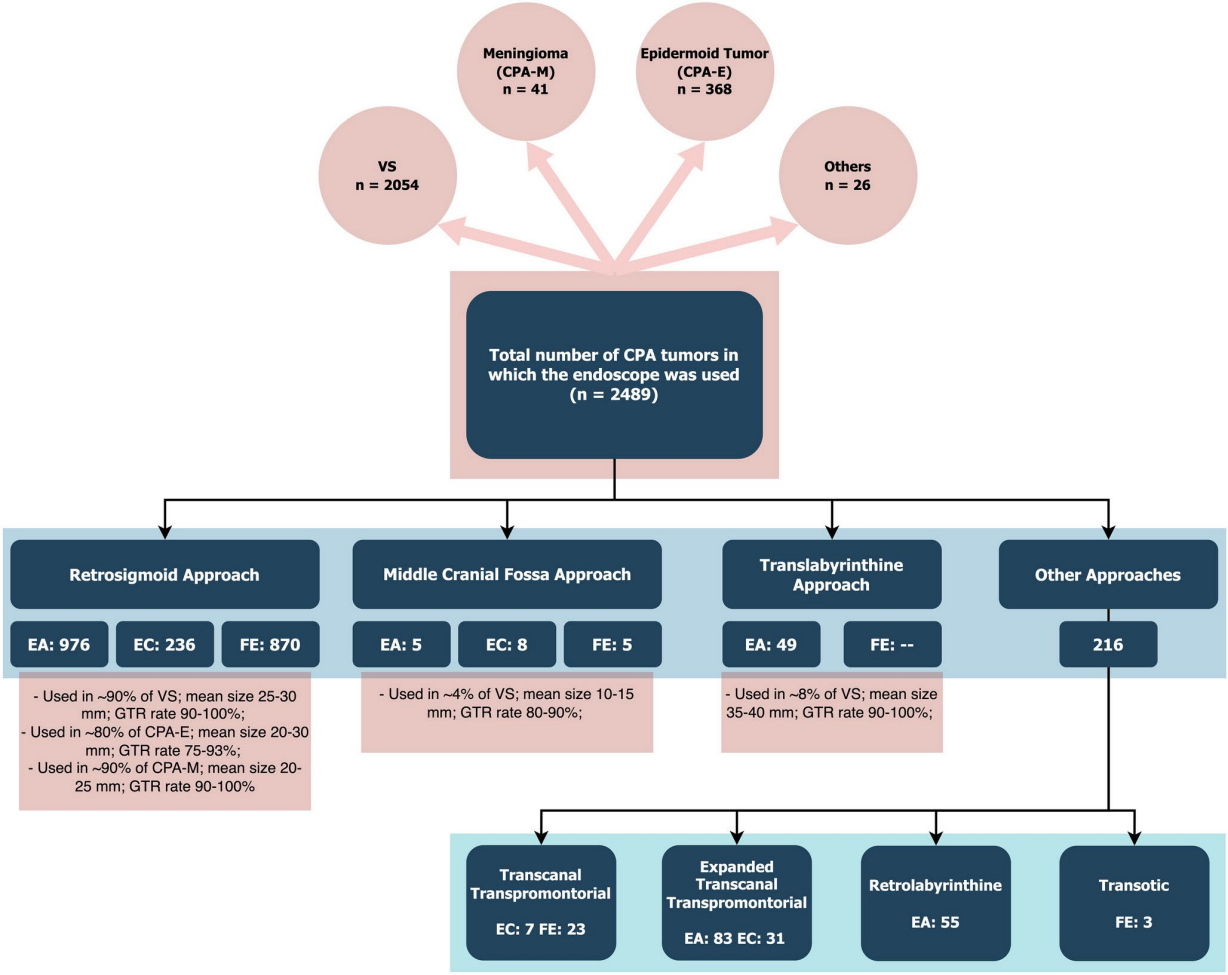
Distribution of collected patients (both case reports and case series regardless from total number) divided by pathology and type of approach. Dividing by surgical approach, there is a mismatch of the total number of patients because few articles did not specify the type of approach. The absence of correspondence among the other approaches is justified by the use of the pterional or the subtemporal approaches that were not reported in the scheme

Distribution by pathology and by approach is described in Fig. 2.

### Vestibular schwannoma

Vestibular schwannoma was the most frequently encountered pathology among the selected studies. A variety of corridors have been employed to access the VS, aiming to maximize tumor resection while simultaneously preserving function and reducing the invasiveness of the procedure in terms of craniotomy size, cerebellar retraction, and, consequently, incidence of postoperative complications and length of hospital stay. The retrosigmoid approach was the most frequently employed, both in the endoscopic-assisted group (931 cases), in the endoscope-controlled group (131 cases), and in the full-endoscopic group (716 cases). The use of angulated microscopic instruments under direct endoscopic visualization was attempted in the middle cranial fossa approach (8 EC and 5 FE) and in the retrolabyrinthine approach (10). On the other hand, the translabyrinthine approach (49) was employed with the endoscope serving solely as a visual assistant at the conclusion of the surgical procedure. Other routes have been explored by otologists, particularly the transotic, transpromontorial, transcanal transpromontorial, and expanded transcanal transpromontorial routes: these were performed with either endoscopic assistance (83), endoscope-controlled (38) or full endoscopic techniques (26) (Fig. 2). Studies comprising ≥ 5 patients have been subjected to more rigorous analysis (Table 1) [6–31]. Only 19.2% of the selected studies were performed under a full endoscopic retrosigmoid approach, while 26.9% used the enhanced visualization offered by the endoscope (EC) to remove tumor remnants in the IAC. Since in most series the endoscope was used only for inspection (without the need for bimanual dexterity) or for limited parts of the surgical procedure (such as checking the IAC for residuals), most authors preferred a free-hand use with a second surgeon dynamically moving the endoscope, while 6 series exclusively used an endoscope holder and 2 tested both free-hand technique and the endoscope holder. For 8 articles, this information was unavailable.

**Table 1:** Cohorts of patients (≥ 5) affected by vestibular Schwannoma underwent surgery by the only means or by auxiliary use of an endoscope (full-endoscopic, endoscope-controlled or endoscope-assisted, FE, EC and EA, respectively)

Authors, year	Study Period	Study design	No. of VS	Age (mean and range)	Surgical Approach	EA vs. FE	EH and/or FH	Endoscope dimensions (mm) and Optics	Size (%)	Surgical time (min)	Lenght of stay (day)	GTR rate (%)	Postoperative Facial function^1^ (House-Brackmann Classification) (%)	Hearing Preservation Rate (%) and Classification	Complications (%)
Goksu, 1999	1989–1996	ROCS	32^2^	NA [20–63]	RS	EA (24)	FH	NA 0°, 30°, 70°	10 ≤ 20 mm (41.7) 6 ≤ 40 mm (25.0) 8 > 40 mm (33.3)	NA	NA	96.8	15 HB1 (62.5) 6 HB2 (25.0) 2 HB3 (8.3) 1 HB4 (4.2)	NA	1 CSF-leak (4.2%)
						EC (8)			13.7 mm (mean)			100	8 HB1 (100)	4 out of preop serviceable hearing (50) (PTA change < 10dB and SDS < 15%)	0
Wackym, 1999	1993–1999	ROCS	78	50.3 y [14–76]	RS (68) TL (7) MCF (3)	EA	EH and FH	2.7 and 4 0°, 30°	9 Intracanalicular (11.5) 27 ≤ 15 mm (34.6) 35 ≤ 30 mm (44.9) 7 ≥ 30 mm (9.0)	NA	NA	93.6	NA	NA	1 Local wound infection 1 Meningitis 1 cerebellar infarction
King, 1999	1996–1997	ROCS	10	49 y [37–76]	RS	EA	EH	2.7 and 3.7 0°, 30°	1 Samii T2 (10.0) 4 Samii T3a (40.0) 3 Samii T3b (30.0) 2 Samii T4a (20.0)	NA	NA	90.0	9 HB1-2 (90.0) 1 HB4 (10.0)	NA	0
Magnan, 2002	1993–1998	ROCS	119	NA	RS	EA	FH	4 and 1.7 0°, 30°	20 Intracanalicular (16.8) 71 ≤ 10 mm (59.7) 28 ≤ 20 mm (23.5)	NA	8	98.5	110 HB1 (92.4) 5 HB2 (4.2) 2 HB3 (1.6) 1 HB4 (0.8)	11 Intracanalicular (52) ≤ 10 mm (49) ≤ 20 mm (42) Shelton Classification	10 CSF-leak (8.4%)
Yuguang, 2004	1996–2003	ROCS	8	NA [23–57]	RS	EA/EC	EH	6 0°, 30°	20–45 mm (range)	35–125 microneurosurgery 10–30 Neuroendoscopy	NA	100	NA	NA	0
Goksu, 2005	1996–2002	ROCS	60	51.1 y [24–70]	RS	EA	NA	NA 0°, 30°, 70°	28 < 20 mm (46.7) 27 < 40 mm (45) 5 ≥ 40 mm (8.3)	NA	NA	100	41 HB1 (68.3) 16 HB2-4 (26.7) 2 HB5 (3.3) 1 HB6 (1.7)	11 out of 45 preop AAO-HNS A-B (24.4)	8 CSF-leak (13.3) 1 Meningitis (1.7)
Gerganov, 2005	2001–2004	POCS	18	49.4 y [34–67]	RS	EA	NA	NA NA	39 mm (mean)	NA	NA	61	7 HB ≤ 3 (39) 11 HB > 3 (61)	NA	NA
Kabil, 2006^3^	2001–2005	ROCS	112	56.0 y [32–68]	RS	FE	EH	2.7 and 4 0°, 30°	26 mm (mean) 6–57 mm (range)	132	2.2	94.6	97 HB1 (86.6) 9 HB2 (8.0) 6 HB3 (5.4)	59 out of 101 preop serviceable/some hearing (< 50 dB loss or < 80 dB loss, respectively) (58)	3 CSF-leak (2.7) 1 Hydrocephalus (0.9)
Hori, 2006	2004–2005	ROCS	33	50.0 y [35–65]	RS	EC	EH	4 0°, 30°, 70°	3 Koos 1 (9.1) 8 Koos 2 (24.2) 7 Koos 3 (21.2) 15 Koos 4 (45.5)	NA	NA	84.4	NA	8 out of 16 of preop Gardner-Robertson’s class I-II (50)	1 endoscopic mechanical injury (3.0)
Gerganov, 2009^4^	NA	POCS	30	45.6 y [18–72]	RS	EA	FH	2 0°, 30°	5 Samii T1 (16.7) 4 Samii T2 (13.3) 6 Samii T3a (20.0) 8 Samii T3b (26.7) 4 Samii T4a (13.3) 3 Samii T4b (10.0)	NA	NA	100	16 HB1 (53.3) 5 HB2 (16.7) 3 HB3 (10.0) 3 HB4 (10.0) 2 HB5 (6.7) 1 HB6 (3.3)	Not data on useful hearing preop (Hannover Classification)	1 CSF-leak (3.3)
Shahinian, 2011	2001–2010	ROCS	527	57.0 y [30–71]	RS	FE	EH	2.7 and 4 0°, 30°	28 mm (mean) 3–58 (range)	193	NA	94.12	491 HB1-2 (95.9) 21 HB3-4 (4.1) 15 HB5-6 (2.9)	213 out of 374 preop serviceable/some hearing (57)	17 CSF-leak (3.3) 1 hydrocephalus (0.2) 13 Superficial Wound Infection (2.5) 39 Recurrence/residual (7.6)
Kumon, 2012	2000–2011	ROCS	28	51.8 y [NA]	RS	EA	EH and FH	NA 30°,70°	21.4 mm (mean)	NA	NA	39.3	Yanagihara Grading System	9 out of 18 preop Gardner-Robertson’s Class I-II (50)	3 Recurrence (10.7)
Chovanec, 2012^4^	2008–2010	POCS	39	47.0 y [26–73]	RS	EA	NA	4 0°, 30°, 70°	2 House Grade 1 (< 10 mm) (5.1) 5 House Grade 2 (10–20 mm) (12.8) 9 House Grade 3 (20–30 mm) (23.1) 22 House Grade 4 (30–40 mm) (56.4) 1 House Grade 5 (> 40 mm) (2.6)	NA	NA	97.4	27 HB1 (69.2) 4 HB2 (10.3) 8 HB3 (20.5)	8 out of 26 preop Gardner-Robertson’s Class I-II (30.7)	20 Pseudomeningocele (51) 3 non-incisional CSF-leak (8)
Iacoangeli, 2013	2009–2011	ROCS	10	52.1 y [29–71]	PSRL	EA	NA	NA NA	2 Samii T2 (20.0) 4 Samii T3a (40.0) 2 Samii T3b (20.0) 1 Samii T4a (10.0) 1 Samii T4b (10.0)	412.5	< 10	80	2 HB1 (20.0) 4 HB2 (40.0) 3 HB3 (30.0) 1 HB4 (10.0)	6 out of 7 preop AAO-HNS grade A-B (85.7)	0
Presutti, 2014^5^	2006–2013	ROCS (Multicenter)	81	52.8 y [19–84]	RS	EA	NA	4 30°, 45°, 70°	31 ≤ 25 mm (38.3) 39 ≤ 40 mm (48.1) 11 > 40 mm (13.6)	NA	NA	100	53 HB1-2 (65.4) 23 HB3-4 (28.4) 5 HB5-6 (6.2)	No preop AAO-HNS grades	6 CSF-leak (7.4) 3 PCF Hemorrhage (3.7) 1 Meningitis (1.2)
Setty, 2015	2006–2013	POCS	12	46.7 y [26–68]	RS	FE	EH	NA 0°, 30°	7 ≤ 15 mm (58.3) 5 ≤ 20 mm (41.7)	261	4	91.6	11 HB1 (91.7) 1 HB3 (8.3)	8 out of 11 preop AAO-HNS A-B (72.7)	0
Marchioni, 2018	2015–2017	ROCS	20	53.4y [22–76]	ExpTT	EA	NA	4 0°	16 Koos 2 (80.0) 4 Koos 3 (20.0)	205 [145–248]	5	100	17 HB1 (85.0) 3 HB2 (15.0)	NA	0
Marchioni, 2019	2015–2018	ROCS	112	NA	RS (18)	EA	FH	4 0°	26 Koos 1 (23.2) 34 Koos 2 (30.4) 28 Koos 3 (25.0) 24 Koos 4 (21.4)	NA	NA	77.8 RS	86 HB1 (76.8) 12 HB2 (10.7) 7 HB3 (6.3) 5 HB4 (4.5) 2 HB5 (1.8)	NA	7 CSF-leak (6.3)
					TL (41)							100 TL			
					ExpTT (47)							91.8 ExpTT			
					TO (4)							100 TO			
					RL (1)							100 RL			
					MCF (1)							100 MCF			
Corrivetti, 2019^6^	2017–2019	ROCS	32	50.2 y [25–76]	RS	EA	FH	4 0°, 45°, 70°	4 < 10 mm (12.5) 15 11–20 mm (46.9) 12 21–30 mm (37.5) 1 31–40 mm (3.1)	NA	NA	84	29 HB1 (90.6) 2 HB2 (6.3) 1 HB4 (3.1)	8 out of 11 preop AAO-HNS A-B (81.8)	1 CSF-leak (3.1)
Caballero-García, 2021^7^	2016–2020	ROCS	31	49.4 y [23–64]	RS	FE	FH	4 0°, 30°, 45°	4 Samii T3a (12.9) 4 Samii T3b (12.9) 6 Samii T4a (19.4) 17 Samii T4b (54.8)	286.5^7^	7.5^7^	92.50	25 HB ≤ 3 (80.7) 6 HB > 3 (19.3)	5 out of 8 preop Gardner-Robertson’s Class I-II (65.5%)	1 CSF-leak (3.2)
Jia Xian-Hao, 2022^4^	2019–2020	ROCS	7	45.7 y [24–63]	MCF	EA/EC	FH	3 70°	2 < 10 mm (28.6) 3 < 15 mm (42.9) 2 < 20 mm (28.6)	NA	NA	84.2	6 HB1 (85.7) 1 HB3 (14.3)	2 out of 3 preop AAO-HNS A-B (66.7)	0
Yunke Bi, 2022	2019–2020	ROCS	61	47.0 y (14.6 SD)	RS	EA/EC	FH	2.9 0°	18 Koos 2 (29.5) 12 Koos 3 (19.7) 31 Koos 4 (50.8)	NA	NA	98.4	50 HB1 (81.9) 5 HB2 (8.2) 4 HB3 (6.6) 2 HB4 (3.3)	NA	NA
Yang S, 2023	2019–2022	ROCS	45	NA [35–77]	RS	EA/EC	NA	NA 30°	NA	NA	NA	91.1	20 HB1 (44.4) 18 HB2 (40.0) 3 HB3 (6.8) 2 HB4 (4.4) 2 HB6 (4.4)	No classification (66.7)	NA
Yang Z, 2023	2019–2022	ROCS	16	54.6y [33–75]	RS	EA/EC	EH	4 30°	44.7 mm (mean) 36–62 (range) 16 Koos 4	NA	NA	100	5 HB1 (31.3) 6 HB2 (37.5) 3 HB3 (18.8) 2 HB5 (12.5)	NA	2 CN VII damage (12.5) 1 Hematoma (6.25)
Hosoya, 2023	2019–2022	ROCS	33	47.3y [33.6–61]	RL	EA	NA	NA 30°	17.4 mm ± 5.7 4 Koos 1 20 Koos 2 8 Koos 3 1 Koos 4	NA	NA	54.6	33 HB1	26 out of 33 No classification for auditory function (79%)	0
Zhang, 2024	2014–2023	ROCS	7	57.6 y (7.7 SD)	RS	FE	FH	4 0°, 30°, 45°, 70°	24.4 mm (mean) 13–50 (range) 3 Koos 2 (42.9) 2 Koos 3 (28.6) 1 Koos 4 (14.3) 1 Koos 5 (14.3)	252.9 ± 61.5	10.3 ± 1.5	71.4	3 HB2 (42.9) 4 HB3 (57.1)	6 out of 6 No classification for auditory function (100%)	0

ROCS: retrospective observational case series; POCS: prospective observational case series; RS: retrosigmoid approach; TL: translabyrinthine approach; MCF: middle cranial fossa approach; PSRL: presigmoid retrolabyrinthine; TT: transcanal transpromontorial; ExpTT: Expanded Transcanal Transpromontorial; TO: transotic; RL: retrolabyrinthine; EA: endoscope-assisted; FE: fully endoscopic; EH: endoscope holder; FH: free hand; GTR: gross total resection; HB: House-Brackmann; AAO-HNS: American Academy of Otolaryngology-Head and Neck Surgery; PCF: posterior cranial fossa; CN: cranial nerve; CSF: cerebrospinal fluid

^1^ The reported facial nerve function was the one at the last follow-up, if available

^2^ Endoscopic inspection was not performed in one patient affected by NF2. In 24 patients the endoscope was used only for inspection (EA), while in 8 patients (mean VS size 13.7 mm) the removal was endoscope-controlled (EC). GTR was obtained in 100% of cases without damage of FN (100% HB1) but with auditory function preservation only in 4 despite the anatomic preservation of the cochlear nerve in all cases

^3^ Authors stated that in case of evidence of residual in the lateral portion of the IAC, tumor dissection was performed under endoscope guidance (EC). This operation was always performed for small tumors (n. 28)

^4^ The study compared two groups. Only patients underwent endoscopic-assisted resection were considered in the current analysis

^5^ The clinical evaluation of facial nerve function was based on the “rough” facial nerve grading system proposed by “Alicandri-Ciufelli, M., Piccinini, A., Grammatica, A., Salafia, F., Ciancimino, C., Cunsolo, E., Pingani, L., Rigatelli, M., Genovese, E., Monzani, D., Gioacchini, F. M., Marchioni, D., & Presutti, L. (2013). A step backward: the ‘Rough’ facial nerve grading system. Journal of cranio-maxillo-facial surgery: official publication of the European Association for Cranio-Maxillo-Facial Surgery, 41(7), e175–e179. 10.1016/j.jcms.2012.11.047”

^6^ The endoscopic assistance was performed with flexible endoscope in 26 cases

^7^ The results referred to all CPA lesions in their series, where others tumor types were reported, such as meningioma, epidermoid cyst, cholesteatoma and metastasis

#### Extent of resection and facial nerve outcome in VS

Stratifying by the endoscope use (EA, EC, or FE), no statistically significant differences emerged among studies in obtaining a gross total resection (*p* = 0.68) and a favorable facial nerve outcome (HB ≤ 2) (*p* = 0.89). Considering all 1553 patients, a weighted average of 92.6% was obtained for GTR with 5.15% of complications (4.25% CSF-leak, 0.32% infections, 0.26% vascular injuries such as bleeding or cerebellar infarction, 0.19% mechanical injuries related to the endoscope and 0.13% hydrocephalus).

Although a clear heterogeneity was evident between studies - in an attempt to compare them − 16 articles [6–10, 13, 14, 17–25] presented their results subgrouping the VS by size (while 9 articles reported only means and another 5 reported an average size > 20 mm): 52.5% of VS were < 20 mm of diameter. However, this measurement was not always specified if it was calculated exclusively on the extrameatal part. Of these 16 articles, only 12 [6, 8, 9, 14, 17–23, 25] individually reported the House-Brackmann grade for the postoperative FN outcome or, at least, divided their patients into groups with favorable (HB 1-2) and unfavorable (HB ≥ 3) outcomes. Over a number of 558 patients, a percentage of 85.7% (weighted average) showed a favorable outcome.

Expanding again the number of studies (not filtering by size) and selecting those that used the HB scale system for grading the FN function (20 studies for a total of 1332 patients), a mean percentage of 90% (weighted average) of favorable HB came out as results.

A summary of the postoperative hearing function was more difficult to calculate, since different classifications have been used throughout the years and among the studies.

**Fig. 3:**
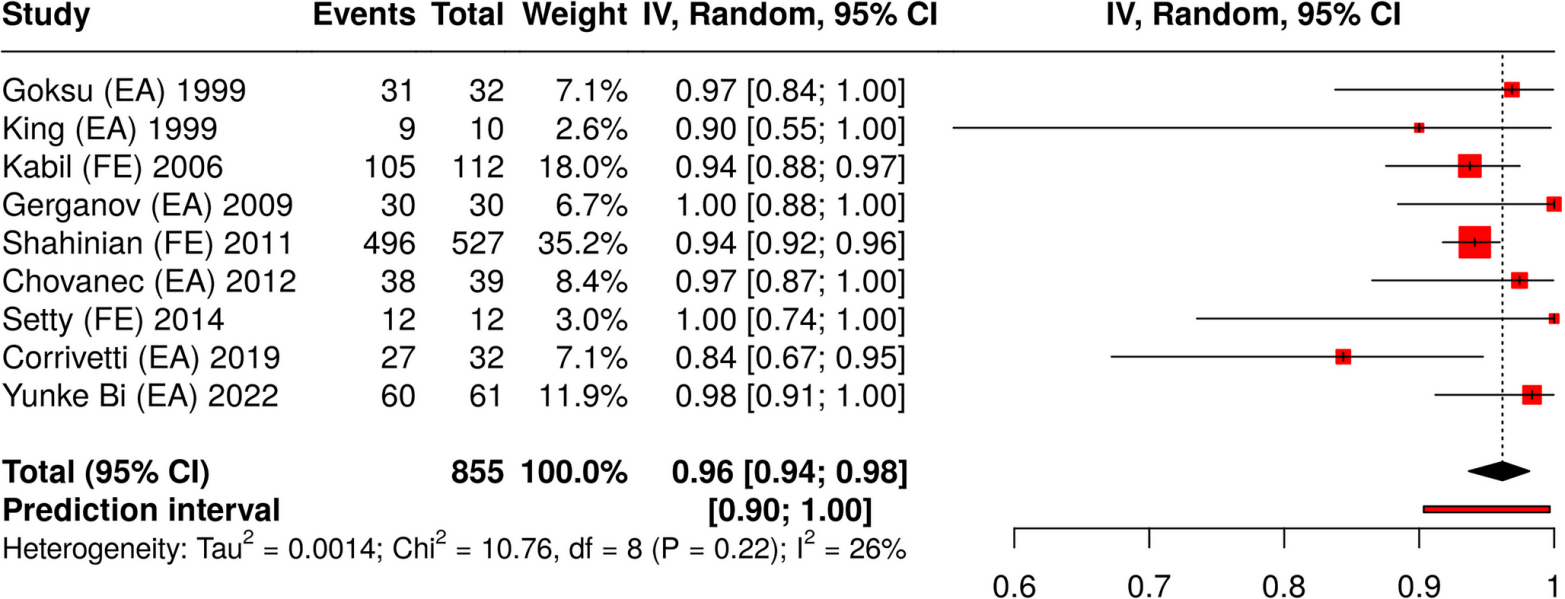
Forest plot (meta-analysis of proportions) for the rate of gross total resection of selected studies with VS patients where either EA or FE were performed. A random effects model with the inverse variance method was used. A DerSimonian-Laird approach was used to calculate the heterogeneity between studies

**Fig. 4:**
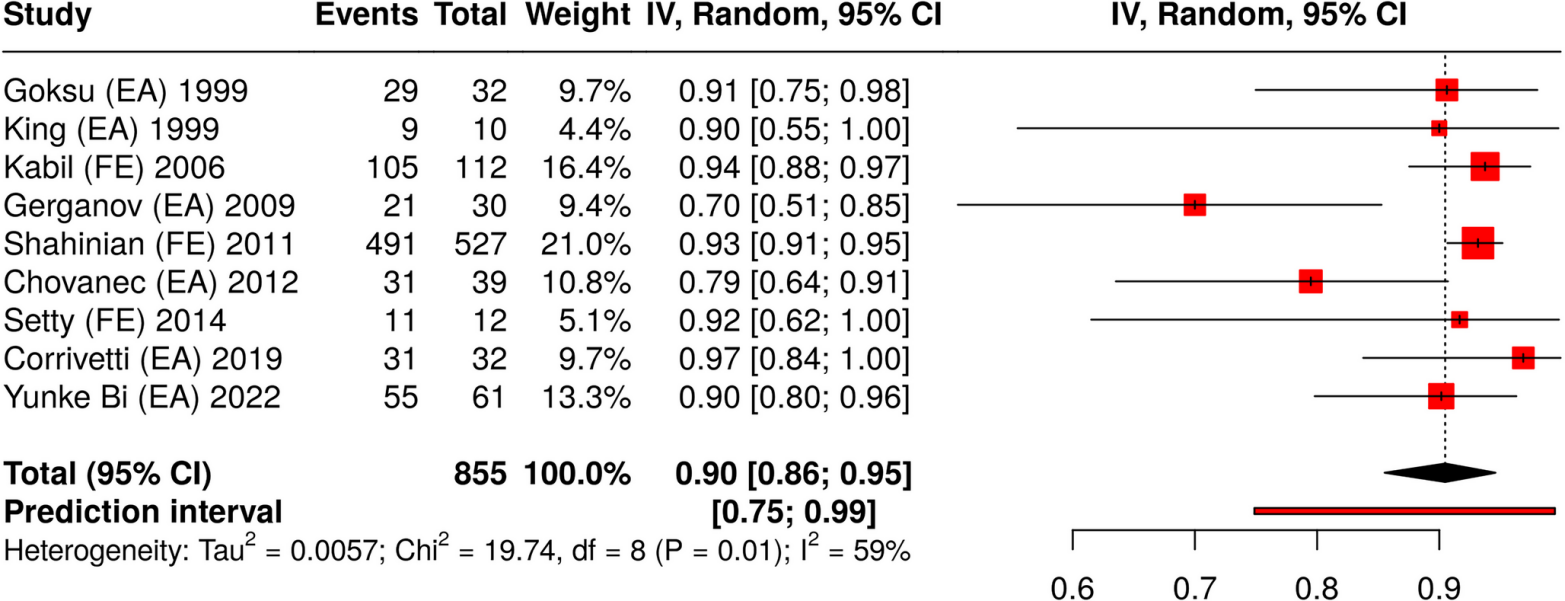
Forest plot (meta-analysis of proportions) for favorable facial nerve outcome (House-Brackmann ≤ 2) of selected studies with VS patients where either EA or FE were performed. A random effects model with the inverse variance method was used. A DerSimonian-Laird approach was used to calculate the heterogeneity between studies

The NOS scale for case-control and single-arm observational studies was applied in order to perform a comparative analysis between studies (Supplementary Table 1). Gross total resection and favorable outcome for FN (HB 1-2) were used as comparative terms to perform a meta-analysis of proportion. All together 9 studies were analyzed with a total of 855 subjects. Based on the analysis performed using a random effects model with the inverse variance method and, the summarized proportion was 0.79 (95%CI 0.78-0.80) for gross total resection (Fig. 3) and 0.77 (95%CI 0.76-0.79) for favorable outcome (Fig. 4). For the latter a significant heterogeneity was detected (*p* = 0.01), suggesting inconsistent effects in magnitude and/or direction. The I^2^ value indicated that 59% of the variability among studies arises from heterogeneity rather than random chance.

### Epidermoid tumor

The endoscope was used in the resection of 368 epidermoid tumors (ET). Excluding case reports, small case series, or articles where information was not enough to categorize and analyze results, 196 patients from 8 articles have been collected (Table 2) [31–39]. The average age at surgery was younger compared to patients affected by vestibular schwannoma. ETs tend to grow and invade contiguous compartments and for this reason 112 ETs were classified as pure CPA lesions while the remaining ones showed an extension in the prepontine cistern, at the level of the foramen magnum, or an extension through the tentorium or at the temporomesial level, or Meckel’s Cave. A retrosigmoid corridor was used in 169 cases. The endoscope assisted the microscopic resection in 96 procedures while 99 were performed full-endoscopic. In most cases the endoscope was used free-hand in either “two surgeons-4 hands” or “one surgeon-2 hands” techniques.

**Table 2:** All patients affected by epidermoid tumor (CPA-E) and cerebellopontine angle cholesteatoma (CPA-C) underwent surgery by the only means or by auxiliary use of an endoscope (full-endoscopic, endoscope-controlled or endoscope-assisted, FE, EC and EA, respectively). All the studies were conducted in a single center unless otherwise specified

Authors, year	Study Period	Study design	No. of CPA-E or -C	Age (mean and range)	Tumor Extension, Location or Size	Surgical Approach	EA vs. FE	EH and/or FH	Endoscope outer diameter (mm) and Optics (degree)	Surgical time (min)	EOR (%)	Complications	Length of stay	Complication related to the endoscope	FU (mean and range)
Schroeder, 2004	1994–2002	ROCS	8	36y [11–62]	2 CPA; 1 CPA-prepontine; 3 CPA-temporomesial; 2 CPA-Meckel cave	5 RS; 3 pterional	4 EA 4 EC	4 FH 4 EH	4 0°, 30°, 70°	NA	3 GTR (37.5) 5 STR (62.5)	2 transient CN worsening; 1 permanent CN worsening	NA	No	46 mos [12–98]
Yuguang, 2004	1996–2003	ROCS	14	NA [23–57]	CPA-C dimension range: 10–70 mm	RS	EA/EC	EH	6 0°, 30°	20–50 Microneurosurgery 20–35 Neuroendoscopy	GTR	None	NA	NA	NA
Safavi-Abbasi, 2008	2000–2004	ROCS	12	47y [24–71]	2 CPA; 7 CPA-prepontine; 2 CPA-supratentorial; 2 CPA-bilateral	11 RS; 1 Subtemporal	12 EA	12 FH	NA NA	NA	9 GTR (75.0) 3 STR (25.0)	1 CSF leak, 1 Hydrocephalus, 6 CN worsening	NA	No	27 mos [8–50]
Peng, 2014	2008–2013	ROCS	6	37y [18–59]	3 CPA; 2 CPA-prepontine; 1 CPA-prepontine-Meckel Cave	6 RS	6 FE	6 EH	NA 0°, 30°	NA	5 GTR (83.3) 1 STR (16.7)	3 CN worsening	NA	1 VII-VIII damage	36 mos [14–50]
Tuchman, 2014	2009–2012	ROCS	7	46.7y [23–65]	4 CPA; 1 CPA bilateral-premedullary; 1 CPA-premedullary, prepontine	5 RS; 2 subtemporal	6 EA	6 FH	NA 30°, 45°, 70°	NA	1 GTR (14.3) 2 NTR (28.6) 4 STR (57.1)	1 transient CN worsening; 1 aspetic meningitis	NA	NA	NA
Hu, 2015	2008–2011	ROCS	30	42.4y [NA]	24 CPA; 3 CPA-prepontine-contralateral CPA or foramen magnum; 3 CPA-supratentorial	30 RS	30 FE	30 FH	4 0°, 30°	156.6 ± 28.2	28 GTR (93.3) 2 STR (6.7)	5 fever, 1 communicating hydrocephalus, 2 CN worsening	7.5d ± 2.25	NA	18.4 mos [10–38]
Tan, 2018	2013–2017	ROCS	5	44.0 ± 11.4	CPA-C dimension. 31 mm ± 6.9	RL	EA/EC	FH	4 0°, 30°, 70°	NA	GTR	None	NA	HB1 (11/11); AAO-HNS stability (8/11),	> 12 mos
Vernon, 2022^1^	2006–2016	ROCS	56	NA	14 CPA; 35 CPA-transtentorial extension; 7 CPA-temporomesial extension	49 RS; 7 RS + subtemporal	56 EA	NA	NA NA	NA	NA	NA	NA	NA	NA
Singh, 2022^2^	2010–2019	ROCS	34	31.3y [16–62]	22 CPA; 1 CPA + prepontine; 1 CPA + prepontine + Meckel Cave	34 RS	10 EA 24 FE	34 FH	4 0°, 30°	NA	24 GTR/NTR (NA)	4 Permanent CN worsening; 7 Transient CSF-leak	7.3d [4–14]	NA	48 mos [12–120]
Tammam, 2022	2017–2021	ROCS	16	42.9y [20–80]^3^	16 CPA	NA	16 EA	NA	NA NA	≤ 300	13 GTR (81.3) 3 STR (18.7)	4 CN VII worsening	NA	NA	NA
Zhang H, 2024	2014–2023	ROCS	34	42.6y [NA]	23 CPA; 4 CPA-supratentorial extension; 4 CPA-foramen magnum extension; 1 CPA-prepontine; 2 multicompartimental	34 RS	34 FE^4^	34 FH	4 0°, 30°, 45°, 70°	217.1 ± 90.7	23 GTR (67.7) 7 STR (20.6) 4 PR (11.7)	4 symptoms worsening	14.4d ± 6.3	No	NA [2–12]

CPA-E: cerebellopontine angle epidermoid; ROCS: retrospective observational case series; POCS: prospective observational case series; RS: retrosigmoid approach; RL: retrolabyrinthine approach; EA: endoscope-assisted; FE: fully endoscopic; EH: endoscope holder; FH: free hand; EOR: extent of resection; GTR: gross total resection; NTR: near total resection; STR: subtotal resection; PR: partial resection; CSF: cerebrospinal fluid; CN: cranial nerve; FU: follow-up

^1^ A large cohort of patients treated with endoscopic assistance was reported, but no specifications regarding extent of resection or complications related to the use of the endoscope have been made

^2^ Age, tumor extension, EOR, LOS and complications refer to the group of patients who were treated by a full-endoscopic approach

^3^ Values referred to all patients (32)

^4^ 16 procedures were performed using a two-surgeons four-hands technique and 18 procedures using the one-surgeon two-hands technique

#### Extent of resection in ET

With the exception of the initial report by Schroeder et al. [35] where was not possible to extrapolate if the GTR was obtained for EA or FE procedures and in the series of Singh et al. [36] where GTR and NTR were considered equivalent in the comparison between FE and microscopic resection, the rate of GTR for the included studies was always over 65%. Most authors intended GTR as the complete removal of the capsule, whereas the persistence of the latter was considered a Near Total Resection (NTR). It is notable that, in both cases, there is no restriction on postoperative diffusion-weighted imaging as defined. The extent of resection reached values over 90% in two series where a whole course endoscopic (FE) resection was performed, without increasing the rate of mechanical injuries of neurovascular structures [32, 36]. Singh et al. [36] compared the results of FE to microscope-only resection: no significant differences in EOR or worsening of cranial nerves function were highlighted starting from two similar groups of ET in terms of extension through compartments. Interestingly, a significantly less long length of stay was noted for patients who underwent FE resection but at the cost of an increased risk of postoperative transient CSF leak (29.1% vs. 4.8% in the FE and microscope-only cohorts, respectively). Among the patients who underwent FE resection, 2 were recurrences. The authors were able to reach a near-total resection even in these situations. Among complications, only 1 was clearly related to the endoscope use.

### Meningioma

Only two series, with a total of 11 cases, have been described so far (Table 3) [40, 41]. In seven cases, a full-endoscopic approach was performed to attempt the removal of small meningiomas (mean diameter around 1.55 cm) in the proximity of the IAC and the related neurovascular structures. A complete removal of the lesion was reported by both authors without any worsening of the facial nerve function. In the larger series by Setty et al. [41], the cochlear function was analyzed as well, resulting in no change for the majority of patients, while 2 improved the auditory function and only one experienced a worsening on American Academy of Otolaryngology - Head and Neck Surgery (AAO-HNS) scale. Although different locations of meningiomas were investigated, Schroeder et al. [40] in their series of skull base meningiomas, found a benefit from the endoscope use in detecting and removing tumor tissue in about 50% of retrosigmoid craniotomies. Nevertheless, it was beneficial in inspecting the internal auditory canal, the Meckel’s Cave, or areas behind the jugular tubercle, or beyond the tentorial edges in case of supratentorial extension.

**Table 3:** All patients affected by CPA meningiomas (CPA-M) underwent surgery by the only means or by auxiliary use of an endoscope (full-endoscopic, endoscope-controlled or endoscope-assisted, FE, EC and EA, respectively)

Authors, year	Study Period	Study design	No. of CPA-M	Age (mean and range)	Tumor Extension, Location or Size	Surgical Approach	EA vs. FE	EH and/or FH	Endoscope outer diameter (mm) and Optics (degree)	Surgical time (min)	Simpson Grade or EOR	Complications	Length of stay	Postoperative CNs function	FU (mean and range)
Schroeder, 2011	2002–2009	ROCS	4	53.3y [46–66]	1.57 cm [1.4–1.9]	RS	EA/EC	Both	2.7 and 4 0°, 30°, 45°, 70°	NA	GTR 4/4	1 CSF leak	NA	Diplopia, vertigo, tinnitus	72.9 mos [31–101]
Setty, 2014	2006–2013	ROCS	11	56.9y [31–72]	1.54 cm [0.5–2.5]	RS	FE	FH	4 0°, 30°	166 [122–207]	Simpson grade 2	1 wound infection	3.1 [2–6]	HB1 (11/11); AAO-HNS stability (8/11), improvement (2/11), worsening (1/11)	NA

ROCS: retrospective observational case series; POCS: prospective observational case series; RS: retrosigmoid approach; EA: endoscope-assisted; EC: endoscope-controlled; FE: fully endoscopic; EH: endoscope holder; FH: free hand; GTR: gross total resection; HB: House-Brackmann; AAO-HNS: American Academy of Otolaryngology-Head and Neck Surgery

## Discussion

The evolution of endoscopic techniques has been marked by their integration with traditional microsurgical methods, followed by the possibility of performing all the surgery uniquely with the endoscope. Since the introduction of the concept of endoscope-assisted surgery [42], the potential of the endoscope in the tumor pathology of the posterior cranial fossa and specifically of the CPA was investigated predominantly in vestibular schwannomas [2].

### Advantages of the endoscope use in CPA lesions

**Fig. 5:**
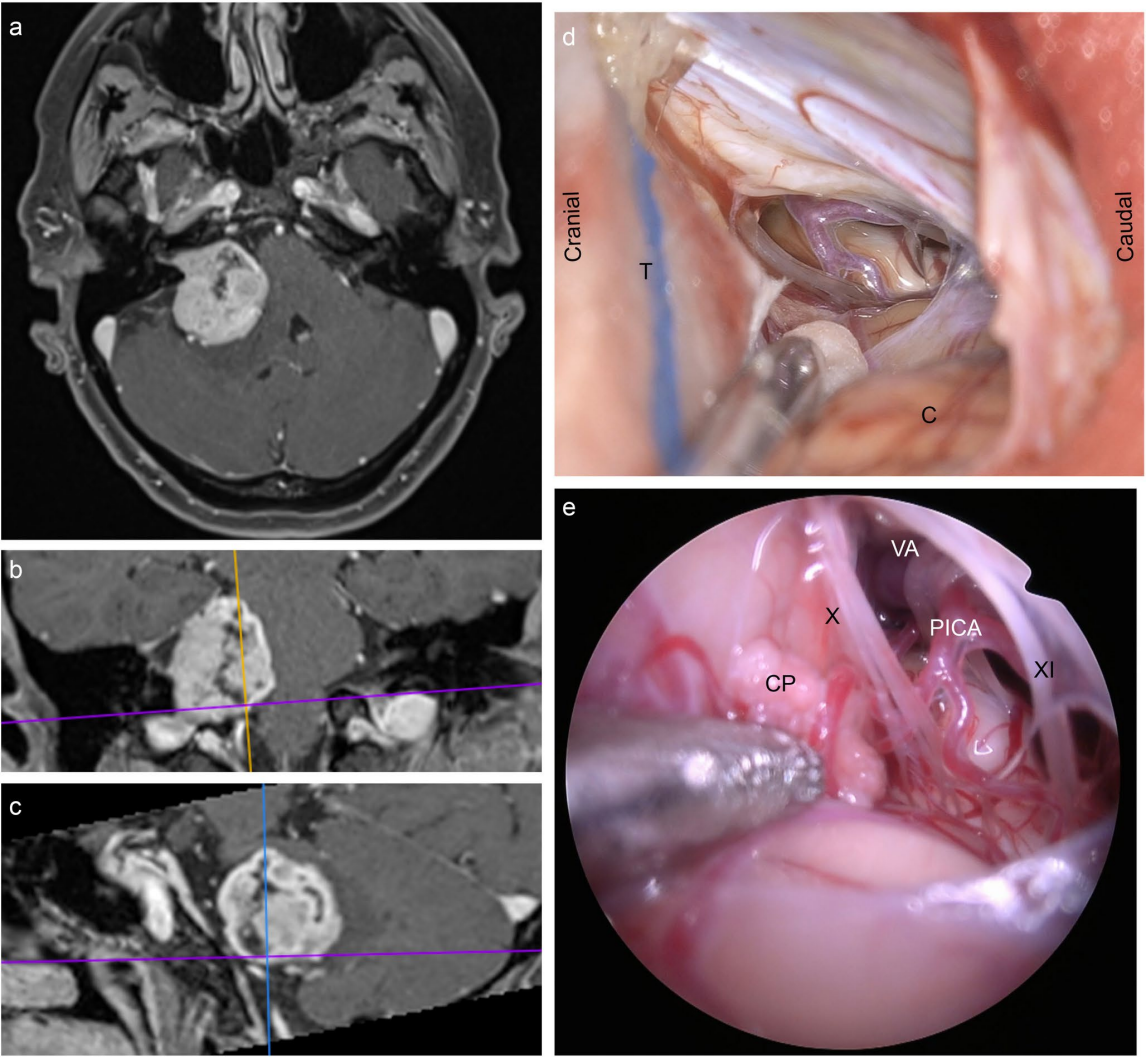
Different point of views: microscopic/exoscopic vs. endoscopic views. Preoperative contrast-enhanced T1-weighted MRI of a right Samii 4a vestibular schwannoma (axial cut, **a**); coronal and sagittal cuts (**b** and **c**, respectively) show the inferior compartment and the vascular relationships which were evaluated immediately after cistern opening with either the exoscope (**d**) and the endoscope (**e**); **d**, the inferior pole of the tumor (T) is covered with a cotton, the suction retracts the cerebellum (C), and the line of sight of the exoscope points to the lateral cerebellomedullary cistern, viewing part of the roots of the tenth cranial nerve and a vessel passing over the lateral surface of the medulla oblongata can be seen; e, a better anatomical detail is obtained with the direct light of a 30° rigid endoscope (Olympus), highlighting the choroid plexus immediately under the suction, the tenth cranial nerve’s rootlets (X), the eleventh cranial nerve (XI), the collateral rami of the posterior inferior cerebellar artery (PICA) and its medial origin from the vertebral artery (VA)

One of the primary benefits of endoscopy was the ability to offer enhanced visualization with a direct, wide, and panoramic view of the CPA increasing the possibility to identify and preserve the critical neurovascular structures that reside in the area. Compared to the direct light of the operating microscope, the angled optics of the endoscope offered the possibility to look around corners, behind the neurovascular structures, or behind the tumor itself (Fig. 5). This advantage in visualization was immediately clear in the narrow and deep surgical corridors or where the anatomy of the corridor, such as the retrosigmoid one, collided with the straight line of vision, which is offered by the operating microscope, preventing the possibility to look around bony or dural corners (i.e., IAC fundus) [43].

Indeed, different angled optics were used, most commonly 30° and 70°. Since the aim of the endoscope was to look around corner and specifically to look inside the IAC, ideally, the more angled the optics, the greater the possibility of discovering a residual. Hori et al. [13] and Kumon et al. [16], using only angled optics reached a percentage of 84.4% and 39% of GTR, respectively. In the latter study, although the percentage of GTR was low compared to the avarage percentage reported in other studies, the comparison with the microscopic-only approach showed a statistically significant difference in terms of extent of resection, even more when the same comparison was made between those VSs that extended over the mid-portion of the IAC. Conversely, Yunke Bi et al. [25] reported the use of endoscope assistance in their retrospective series of 61 patients but no angled optics were used. In fact, a drill of almost all the posterior wall of the IAC (8–10 mm) was performed in all cases with intracanalicular extension losing what is one of the advantage of endoscope assistance.

Furthermore, an enhanced direct visualization allowed an easier detection of openend mastoid cells reducing the risk of postoperative CSF-leak [6, 44]. Although considered only a relative benefit [11], a direct and early detection of the facial nerve course from its brainstem junction/root entry zone could increase the rate of anatomical and functional preservation.

In accordance with the growing preference for minimally invasive procedures, some authors have explored the use of endoscopy as a sole means of visualization for VS [12], ET [33, 45] and meningiomas [41]. This approach has the potential to reduce the size of the craniotomy and minimize the need for cerebellar retraction. A full-endoscopic approach was possible with the introduction of the endoscope holder which allowed a bimanual surgical technique [46]. However, few authors have pushed and perfectioned a technique that guarantee no need of mechanical holder neither the presence of a second surgeon to hold freehand the endoscope when there was the necessity to perform a bimanual dissection [47, 48], but its feasibility in a retrosigmoid approach has not been assessed.

Nevertheless, others discouraged the endoscopic resection of ET because of the presence of adherences and the fat content that can disturb the endoscopic visualization [34]. Similarly, a frequent need to clean the lens could be required in meningioma resection, due to the bloody nature and the accumulation of bone dust from drilling. Indeed, most authors considered it a reliable tool in the neurosurgeon’s hand to inspect behind blind corners or check for residuals in order to increase the extent of resection. A review of references on VS (*n* = 25, both EA and FE) showed that only 16 (64%) documented the number and size of patients. In series reporting GTR > 90%, almost half of the tumors were under 25 mm [6–10, 14, 20, 49]. However, 90% of GTR was achieved even in the case of larger VSs, both endoscopic-assisted [17, 19, 25] and full-endoscopic [24].

Reviews of endoscopy use in ET management showed > 65% EOR, contradicting initial results from Schroeder et al. [35] and the discouraging recommendation on the exclusive use of an EC removal [34]. Later studies have reported positive results for ET removal with EC or FE, including EOR, length of stay, and new neurological deficits [31, 32, 36]. Most authors agree on the benefits of using endoscopy to visualize beyond blind spots. It may even be used to access other parts of the skull. This could be useful in cases of multicompartmental ET where it might be better than more invasive approaches. Furthermore, direct visualization of the capsule with an endoscope can predict EOR more accurately than magnetic resonance imaging (MRI), because capsule residual may not be visible on MRI [36].

### Limitations of the endoscope use in CPA lesions

A few limitations come with the use of this tool: manipulating the endoscope within an overcrowded and intricate region necessitates dynamic adjustment to compensate for the absence of three-dimensional vision, a challenge that is exacerbated by vision impairment [14, 38, 50]. Furthermore, being a small surgical window, the endoscope can further reduce the surgical freedom resulting in a “sword fighting” between instruments [51]. The lack of habit to this “unnatural” visualization and the absence of three-dimensional perception can make difficult and dangerous the first experiences with this instrument [51]. Recent improvements in devices with the diffusion of 3D high-definition systems could help in the sense of depth, especially for those surgeons with limited experience in endoscopic surgery [52, 53].

In case of frequent need to clean and consequentially removing-introducing the endoscope several times, a higher risk of damaging the surrounding neurovascular structures should be taken into account. For this reason, larger craniotomies (at least 15 mm) and constant guidance under microscopic visualization during maneuvers have been highly recommended [50]. Furthermore, the possibility of damaging those structures that are located behind the tip of the endoscope is a known complication of endoscopic surgery in the CPA [13, 33].

Another concern is the risk of thermal damage caused by the xenon light source used in some endoscopes. This risk necessitates careful temperature management and the use of cooling techniques to prevent tissue injury [14].

The angled and direct view of the endoscope is important as it allows for better recognition of blind spots, increasing the chance of a gross total resection. However, it could not clearly distinguish tumor tissue from structures of the vestibular ganglion or the distal stump of the vestibular nerve, which could impact the cochlear or even the facial nerve [17].

Endoscopic techniques require specialized training and equipment, which restricts their use [54]. This means that they are only used by the few centers with the necessary resources and expertise. Many other centers continue to rely on traditional microscopic methods. The diffusion of the exoscope, which may offer supplementary viewing angles beyond those of the microscope (especially those that due to the type of approach and patient positioning require the maintenance of a difficult posture), may curtail the adoption of the endoscope in centers where it is not customary. Although equipped with more degrees of movement than the microscope, it still does not allow viewing certain hidden angles that the endoscope allows (Fig. 5).

## Limitations

The present review has several limitations related to the selected studies. The studies differed in their methodologies, patient cohorts, and surgical outcomes, making proper comparisons difficult. Many studies lacked standardized reporting, and most were single-center, retrospective analyses with small sample sizes, causing bias. By not setting a time limit, the most recent studies might not be directly comparable to earlier ones, as technology has advanced. Indeed, advances in technology have led to high-resolution, miniaturized rigid endoscopes, and better endoscope holders. However, there are no significant differences in rates of resection, anatomic or functional facial nerve preservation between the late ‘90/early ‘00 and the more recent series. Tumor size influences outcomes from full endoscopic procedures, preferring this approach for small lesions. Another limitation is the inability to provide precise figures for EC resection, as many studies report EC as a possibility when a tumor remnant was identified during inspection (EA). In order to definitively conclude on the real potential of the endoscope, a randomized, multicenter study should be conducted. Randomized controlled trials would provide stronger evidence for the benefits of endoscopy and help establish guidelines for its routine use in CPA tumor surgeries.

## Conclusions

The use of the endoscope in the surgical management of extra-axial tumors of the cerebellopontine angle (CPA) has shown promising results over the past few decades. The enhanced visualization provided by endoscopic approaches, either as an adjunct to microscopic surgery (endoscope-assisted) or as the primary visualization tool (endoscope-controlled or full-endoscopic), offers several advantages. These include improved identification of residual tumors, better anatomical recognition, and a reduction in postoperative complications such as cerebrospinal fluid leaks. Despite these benefits, the widespread adoption of endoscopic techniques remains limited. The majority of neurosurgeons are more familiar with traditional microscopic methods, and the specialized skills required for endoscopic surgery are typically found in only a few centers. Furthermore, the evidence supporting the routine use of endoscopy in CPA tumor surgeries has been limited to a few prospective and case-control studies. In conclusion, the endoscopic visualization for most tumors of the CPA, and especially for multicompartmental ETs and meningiomas, should be considered not in substitution of the operating microscope, but as an additional tool in the surgical armamentarium.

## Electronic supplementary material

Below is the link to the electronic supplementary material.

Supplementary Material 1

Supplementary Material 2
