# Supplementary material for: A systematic review on the role of the endoscope in the surgical management of cerebellopontine angle tumors: is it time to draw the conclusion?
Source: Eur Arch Otorhinolaryngol. 2025 Apr 30;282(11):5445–60. doi: 10.1007/s00405-025-09427-4 (PMC12605435; doi:10.1007/s00405-025-09427-4)
Supplement: Supplementary file 1 — Supplementary Material 1 [file 405_2025_9427_MOESM1_ESM.docx]

**Supplementary Material:** research strings for Pubmed/Medline and Embase databases

Research Strings

Pubmed

1. (Cerebellopontine angle tumors) AND (endoscopic resection) = 101

("neuroma, acoustic"[MeSH Terms] OR ("neuroma"[All Fields] AND "acoustic"[All Fields]) OR "acoustic neuroma"[All Fields] OR ("cerebellopontine"[All Fields] AND "angle"[All Fields] AND "tumors"[All Fields]) OR "cerebellopontine angle tumors"[All Fields]) AND (("endoscope s"[All Fields] OR "endoscoped"[All Fields] OR "endoscopes"[MeSH Terms] OR "endoscopes"[All Fields] OR "endoscope"[All Fields] OR "endoscopical"[All Fields] OR "endoscopically"[All Fields] OR "endoscopy"[MeSH Terms] OR "endoscopy"[All Fields] OR "endoscopic"[All Fields]) AND ("resect"[All Fields] OR "resectability"[All Fields] OR "resectable"[All Fields] OR "resectates"[All Fields] OR "resected"[All Fields] OR "resecting"[All Fields] OR "resection"[All Fields] OR "resectional"[All Fields] OR "resectioned"[All Fields] OR "resectioning"[All Fields] OR "resections"[All Fields] OR "resective"[All Fields] OR "resects"[All Fields]))

1. (vestibular schwannoma) AND (endoscopic surgery) = 196

("neuroma, acoustic"[MeSH Terms] OR ("neuroma"[All Fields] AND "acoustic"[All Fields]) OR "acoustic neuroma"[All Fields] OR ("vestibular"[All Fields] AND "schwannoma"[All Fields]) OR "vestibular schwannoma"[All Fields]) AND ("endoscopy"[MeSH Terms] OR "endoscopy"[All Fields] OR ("endoscopic"[All Fields] AND "surgery"[All Fields]) OR "endoscopic surgery"[All Fields])

1. (posterior fossa skull base meningioma) AND (endoscopic resection) = 37

"posterior"[All Fields] OR "posteriors"[All Fields]) AND ("fossa"[All Fields] OR "fossae"[All Fields] OR "fossas"[All Fields]) AND ("skull base"[MeSH Terms] OR ("skull"[All Fields] AND "base"[All Fields]) OR "skull base"[All Fields]) AND ("meningioma"[MeSH Terms] OR "meningioma"[All Fields] OR "meningiomas"[All Fields]) AND (("endoscope s"[All Fields] OR "endoscoped"[All Fields] OR "endoscopes"[MeSH Terms] OR "endoscopes"[All Fields] OR "endoscope"[All Fields] OR "endoscopical"[All Fields] OR "endoscopically"[All Fields] OR "endoscopy"[MeSH Terms] OR "endoscopy"[All Fields] OR "endoscopic"[All Fields]) AND ("resect"[All Fields] OR "resectability"[All Fields] OR "resectable"[All Fields] OR "resectates"[All Fields] OR "resected"[All Fields] OR "resecting"[All Fields] OR "resection"[All Fields] OR "resectional"[All Fields] OR "resectioned"[All Fields] OR "resectioning"[All Fields] OR "resections"[All Fields] OR "resective"[All Fields] OR "resects"[All Fields]))

1. (posterior fossa epidermoid cyst) AND (endoscopic resection) = 9

("posterior"[All Fields] OR "posteriors"[All Fields]) AND ("fossa"[All Fields] OR "fossae"[All Fields] OR "fossas"[All Fields]) AND ("epidermal cyst"[MeSH Terms] OR ("epidermal"[All Fields] AND "cyst"[All Fields]) OR "epidermal cyst"[All Fields] OR ("epidermoid"[All Fields] AND "cyst"[All Fields]) OR "epidermoid cyst"[All Fields]) AND (("endoscope s"[All Fields] OR "endoscoped"[All Fields] OR "endoscopes"[MeSH Terms] OR "endoscopes"[All Fields] OR "endoscope"[All Fields] OR "endoscopical"[All Fields] OR "endoscopically"[All Fields] OR "endoscopy"[MeSH Terms] OR "endoscopy"[All Fields] OR "endoscopic"[All Fields]) AND ("resect"[All Fields] OR "resectability"[All Fields] OR "resectable"[All Fields] OR "resectates"[All Fields] OR "resected"[All Fields] OR "resecting"[All Fields] OR "resection"[All Fields] OR "resectional"[All Fields] OR "resectioned"[All Fields] OR "resectioning"[All Fields] OR "resections"[All Fields] OR "resective"[All Fields] OR "resects"[All Fields]))

Embase

1. ('cerebellopontine angle tumors'/exp OR 'cerebellopontine angle tumors' OR (cerebellopontine AND ('angle'/exp OR angle) AND ('tumors'/exp OR tumors))) AND ('endoscopic resection'/exp OR 'endoscopic resection' OR (endoscopic AND ('resection'/exp OR resection)) OR 'endoscopic surgery'/exp OR 'endoscopic surgery' OR (endoscopic AND ('surgery'/exp OR surgery))) = 192
2. ('vestibular schwannoma'/exp OR 'vestibular schwannoma' OR (vestibular AND ('schwannoma'/exp OR schwannoma))) AND ('endoscopic resection'/exp OR 'endoscopic resection' OR (endoscopic AND ('resection'/exp OR resection)) OR 'endoscopic surgery'/exp OR 'endoscopic surgery' OR (endoscopic AND ('surgery'/exp OR surgery))) = 254
3. ('posterior fossa skull base meningioma' OR (posterior AND fossa AND ('skull'/exp OR skull) AND ('base'/exp OR base) AND ('meningioma'/exp OR meningioma))) AND ('endoscopic resection'/exp OR 'endoscopic resection' OR (endoscopic AND ('resection'/exp OR resection)) OR 'endoscopic surgery'/exp OR 'endoscopic surgery' OR (endoscopic AND ('surgery'/exp OR surgery))) = 92
4. ('posterior fossa epidermoid cyst' OR (posterior AND fossa AND ('epidermoid'/exp OR epidermoid) AND ('cyst'/exp OR cyst))) AND ('endoscopic resection'/exp OR 'endoscopic resection' OR (endoscopic AND ('resection'/exp OR resection)) OR 'endoscopic surgery'/exp OR 'endoscopic surgery' OR (endoscopic AND ('surgery'/exp OR surgery))) = 28
