# Supplementary material for: A systematic review on the role of the endoscope in the surgical management of cerebellopontine angle tumors: is it time to draw the conclusion?
Source: Eur Arch Otorhinolaryngol. 2025 Apr 30;282(11):5445–60. doi: 10.1007/s00405-025-09427-4 (PMC12605435; doi:10.1007/s00405-025-09427-4)
Supplement: Supplementary file 2 — Supplementary Material 2 [file 405_2025_9427_MOESM2_ESM.docx]

**Supplementary Table 1**: New Castle-Ottawa Scale (NOS) for studies with more than 10 patients affected by vestibular schwannoma.

| **Study** | **Selection** | | | | **Comparability*** | **Outcomes** | | | **Total** |
| --- | --- | --- | --- | --- | --- | --- | --- | --- | --- |
|  | **Representativeness of exposed cohort** | **Selection of nonexposed cohort*** | **Ascertainment of Exposure** | **Outcome not present at the start of the study** |  | **Assessment of outcomes** | **Length of follow-up** | **Adequacy of follow-up** |  |
| Goksu, 1999 | ***** | NA | ***** | ***** | NA | ***** | ***** | ***** | **6** |
| Wackym, 1999 | ***** | NA | ***** |  | NA |  |  |  | **2** |
| King, 1999 | ***** | NA | ***** | ***** | NA | ***** |  |  | **4** |
| Goksu, 2005 | ***** | NA | ***** | ***** | NA |  |  |  | **3** |
| Gerganov, 2005 |  | NA | ***** |  | NA |  | ***** |  | **2** |
| Kabil, 2006 | ***** | NA | ***** | ***** | NA | ***** | ***** | ***** | **6** |
| Hori, 2006 | ***** | NA | ***** | ***** | NA |  |  |  | **3** |
| Gerganov, 2009^†^ | ***** | * | ***** |  | ***** |  | ***** |  | **5**^†^ |
| Shahinian, 2011 | ***** | NA | ***** | ***** | NA | ***** | ***** | ***** | **6** |
| Kumon, 2012^†^ |  | ***** | ***** | ***** |  |  |  |  | **3**^†^ |
| Chovanec, 2012^1^ | ***** | * | ***** | ***** | * | ***** | ***** | ***** | **8**^†^ |
| Iacoangeli, 2013 |  | NA | ***** | ***** | NA |  |  |  | **2** |
| Presutti, 2014 | ***** | NA | ***** |  | NA | ***** |  |  | **3** |
| Setty, 2015 | ***** | NA | ***** | ***** | NA | ***** |  |  | **4** |
| Marchioni, 2019 | ***** | NA | ***** | ***** | NA |  |  |  | **3** |
| Corrivetti, 2019 | ***** | NA | ***** | ***** | NA | ***** |  |  | **4** |
| Caballero-García, 2021 |  | NA | ***** | ***** | NA |  |  |  | **2** |
| Yunke Bi, 2022 | ***** | NA | ***** | ***** | NA | ***** |  |  | **4** |
| Yang, 2023 |  | NA | ***** | ***** | NA | ***** |  |  | **3** |

^*^Considering that most of the selected studies are single arm studies the full applicability of the Newcastle-Ottawa Scale is limited. In particular, two domains, ‘selection of the non-exposed cohort’ and ‘comparability of cohorts on the basis of the design or analysis controlled for confounders’, were excluded from the assessment as they were irrelevant to the single arm studies.

^†^ Case-control study: comparison between endoscopic assisted microsurgery and only microscopic resection of VS.
